# Supplementary material for: The structural basis of the activation and inhibition of DSR2 NADase by phage proteins
Source: Nat Commun. 2024 Jul 23;15:6185. doi: 10.1038/s41467-024-50410-0 (PMC11263360; doi:10.1038/s41467-024-50410-0)
Supplement: Supplementary file 1 — Supplementary information [file 41467_2024_50410_MOESM1_ESM.pdf]

Supplementary Data and Figures for

The structural basis of the activation and inhibition of DSR2 NADase by phage proteins

Ruiwen Wang[1#], Qi Xu[2,3#], Zhuoxi Wu[1], Jialu Li[2,3], Hao Guo[1], Tianzhui Liao[1], Yuan Shi[2,3], Ling Yuan[1] Haishan Gao[2,3], Rong Yang[4*], Zhubing Shi[2,3*], Faxiang Li[1*]

[1] MOE Key Laboratory of Rare Pediatric Diseases, Center for Medical Genetics, School of Life Sciences, Central South University, Changsha, China.

[2] Zhejiang Key Laboratory of Structural Biology, School of Life Sciences, Westlake University, Hangzhou, Zhejiang, China.

[3] Westlake Laboratory of Life Sciences and Biomedicine, Hangzhou, Zhejiang, China.

[4] State Key Laboratory of Developmental Biology of Freshwater Fish, Engineering Research Center of Polyploid Fish Reproduction and Breeding of the State Education Ministry, College of Life Sciences, Hunan Normal University, Changsha, China.

[#] These authors contributed equally to this work

*Correspondence: chinalfx@163.com (F.L.), shizhubing@westlake.edu.cn (Z.S.), rongyang@hunnu.edu.cn (R.Y.)

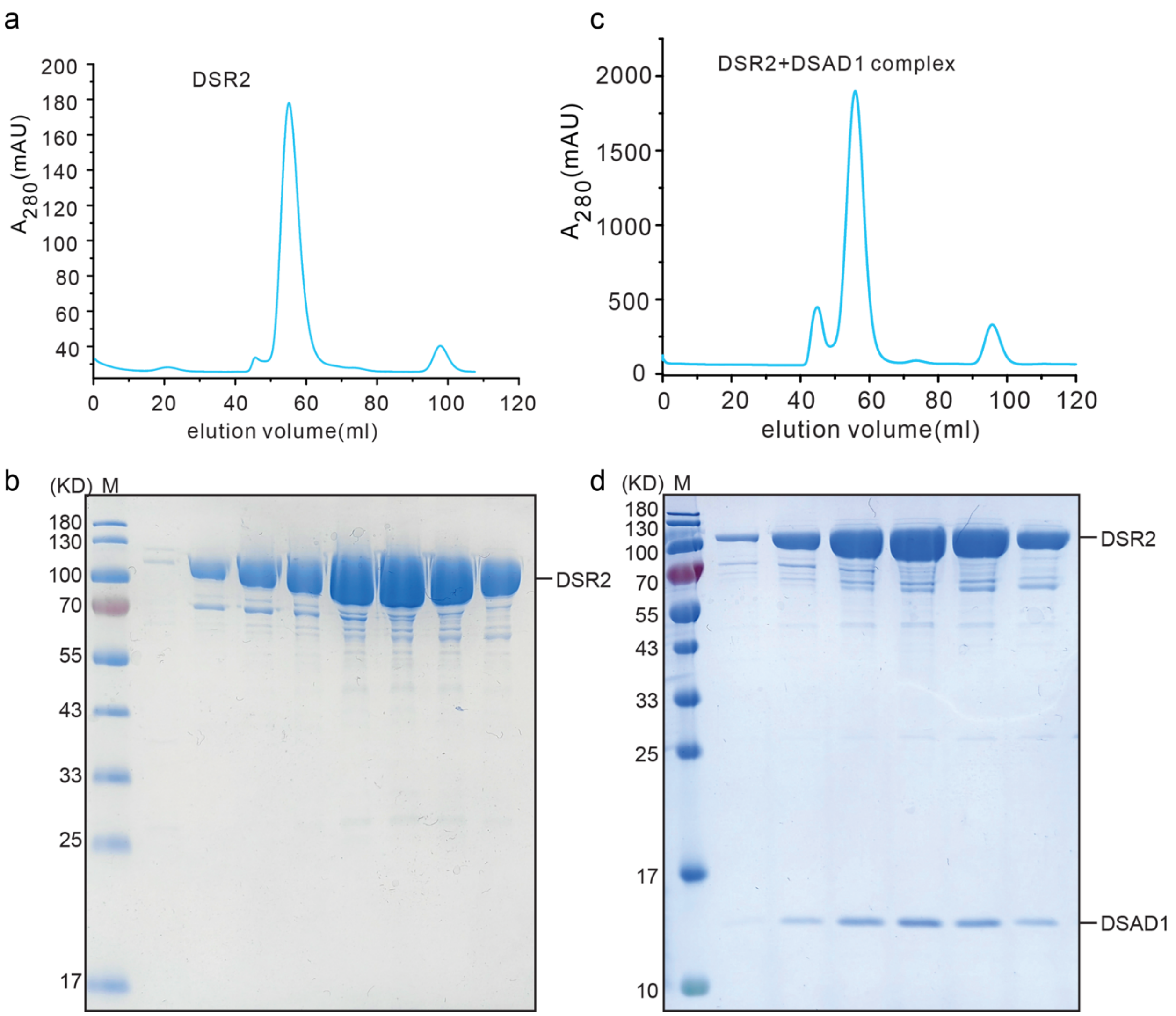

Supplementary Figure 1. Purification of DSR2 and DSR2-DSAD1 complex.

a The gel filtration profile of DSR2 proteins on S200 column. **b** The SDS-PAGE combined Coomassie Brilliant Blue staining showing the protein quality of DSR2. **c** The gel filtration profile of DSR2-DSAD1 complex proteins on UNIONDEX 200PG size exclusion column. **d** The SDS-PAGE combined Coomassie Brilliant Blue staining showing the protein quality of DSR2-DSAD1 complex.

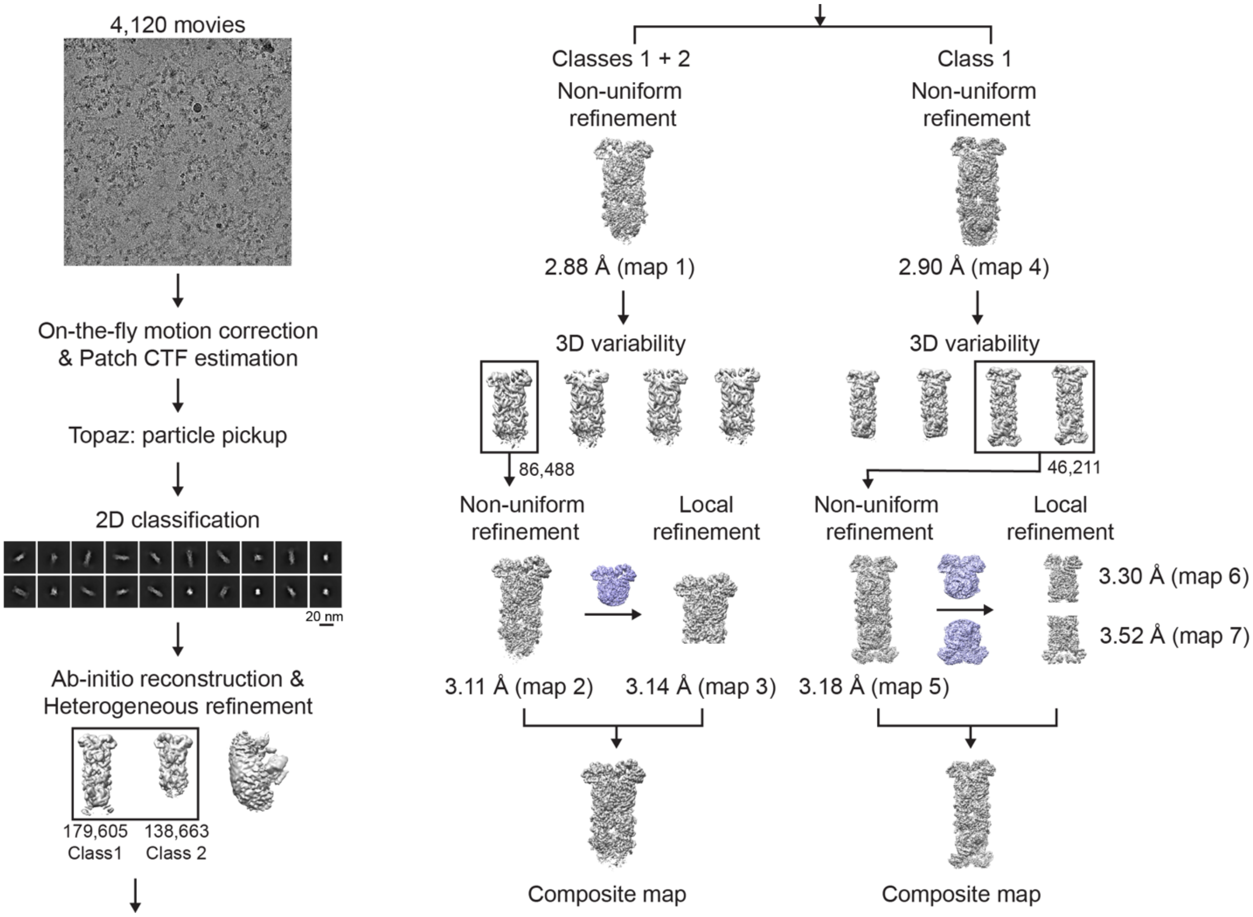

Supplementary Figure 2. Structure determination of DSR2 (H171A) tetramer.

Workflow of the cryo-EM data processing to obtain the 3D reconstructions of DSR2 (H171A) tetramer. Seven cryo-EM maps (labeled maps 1-7) were obtained for the whole structure.

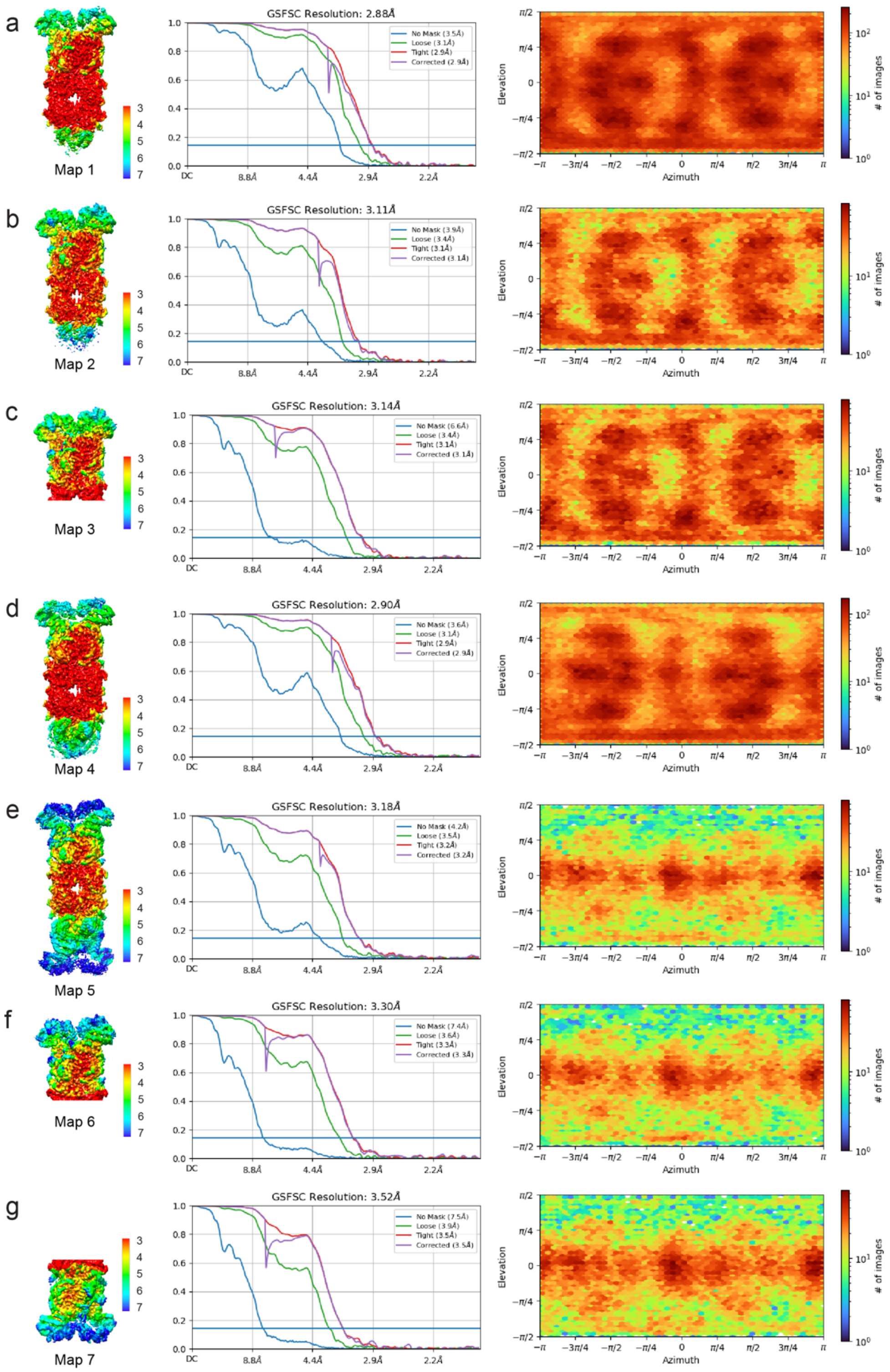

Supplementary Figure 3. The resolution analysis of DSR2 (H171A) tetramer.

a-g Local resolution maps and Fourier shell correlation (FSC) curves of DSR2 (H171A) tetramer. The local resolution maps (**a-g**) correspond to the different cryo-EM maps 1-7 depicted in **Supplementary Figure 2.**

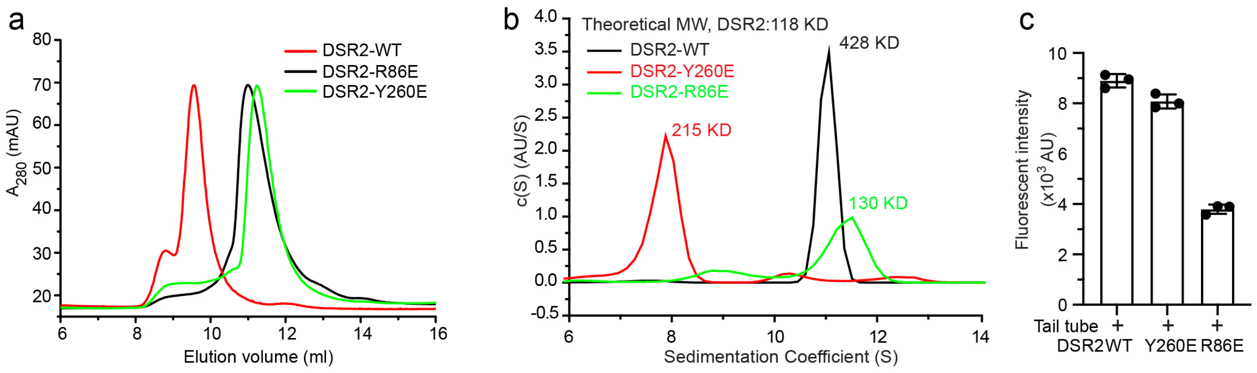

Supplementary Figure 4. The oligomerization state analysis of DSR2 WT and Y260E mutant.

a Gel filtration profiles of WT DSR2 and its R86E, Y260E mutant proteins on the Superdex™ 200 Increase size exclusion column. **b** Analytical ultracentrifugation analysis the molecular weight of the WT DSR2 and R86E, Y260E mutant proteins. **c** NAD$^+$ hydrolase activities of WT DSR2 and its mutants in the presence of TTP.

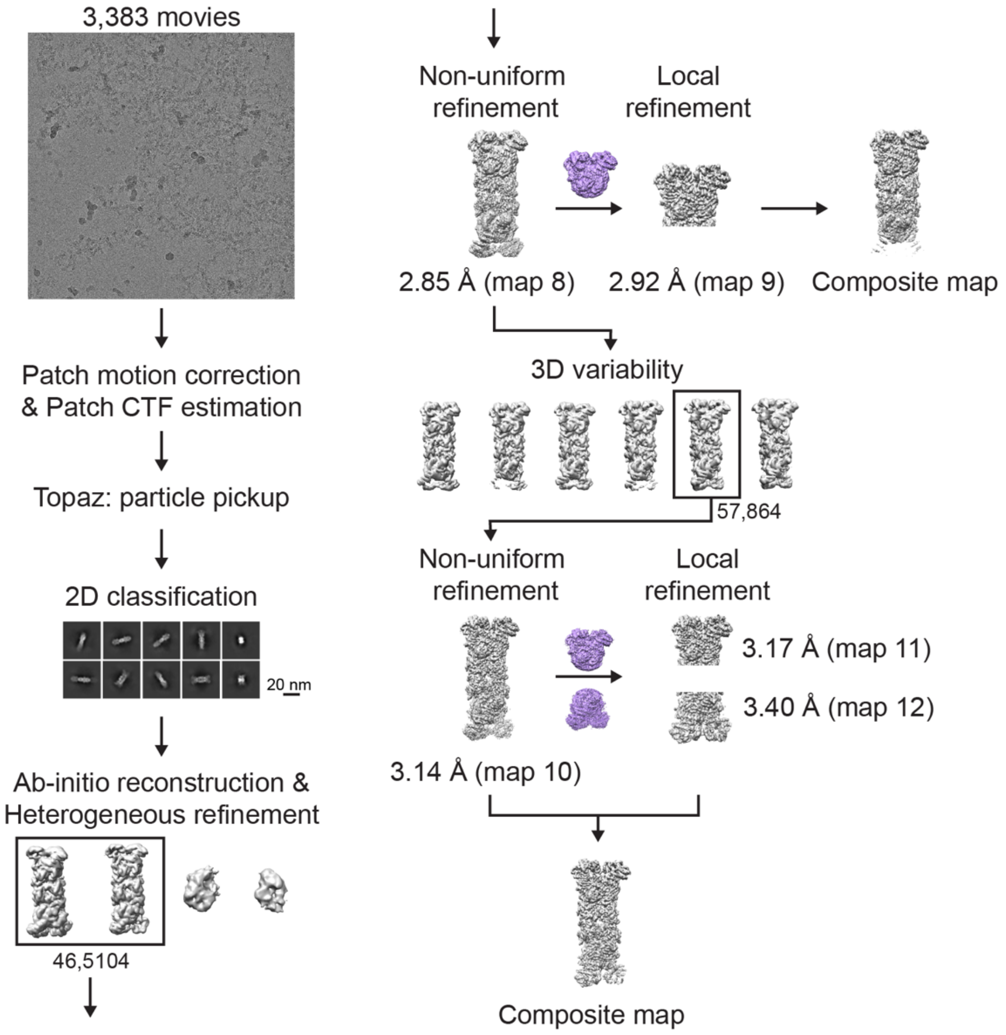

Supplementary Figure 5. Structure determination of DSR2 (H171A)-TTP-NAD$^+$ complex.

Workflow of the cryo-EM data processing to obtain the 3D reconstructions of DSR2 (H171A)-TTP-NAD$^+$ complex. Five cryo-EM maps (labeled maps 8-12) were obtained for the whole structure.

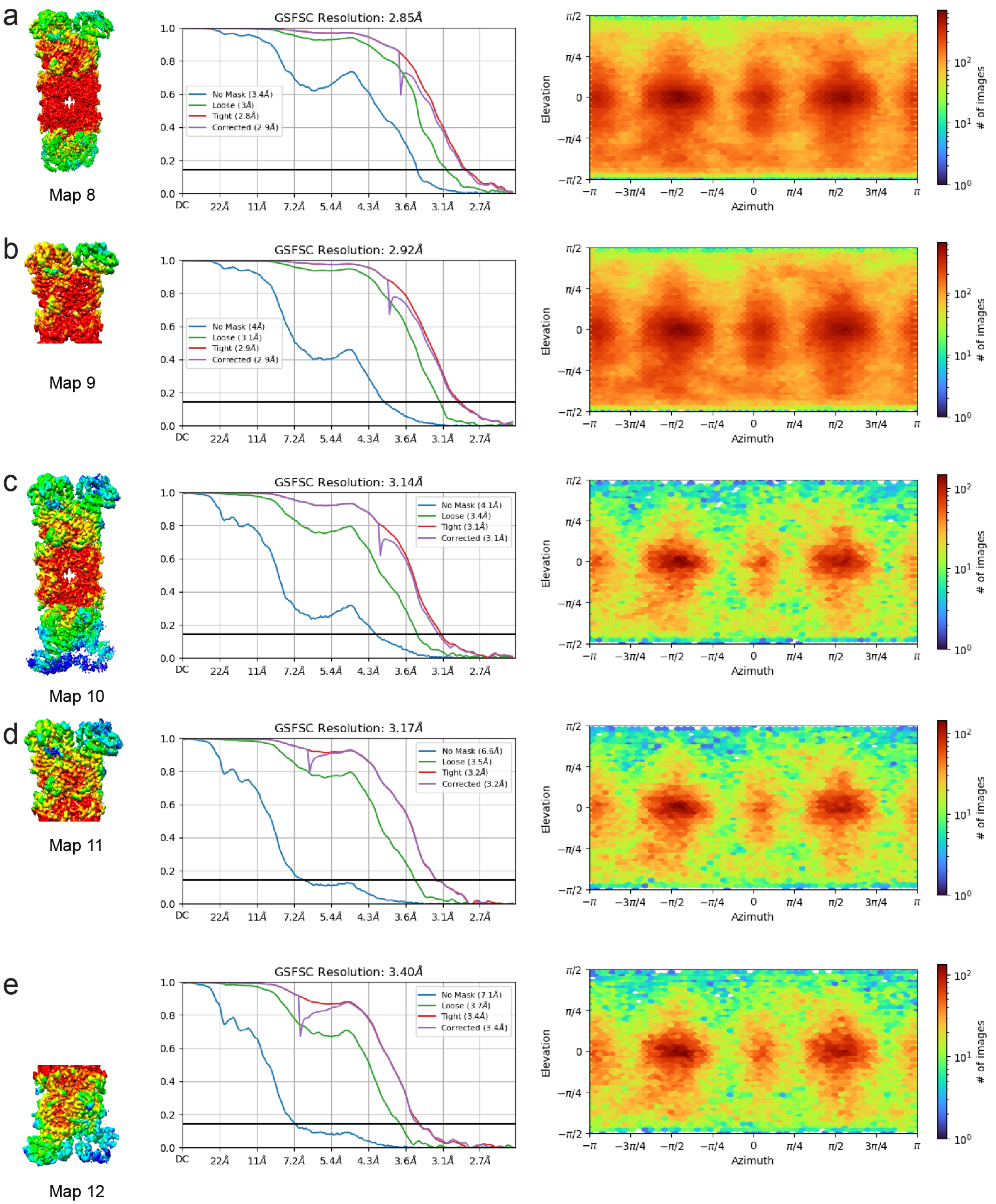

Supplementary Figure 6. The resolution analysis of DSR2 (H171A)-TTP-NAD$^+$ complex.

a-i Local resolution maps and Fourier shell correlation curves of DSR2 (H171A)-TTP-NAD$^+$ complex. The local resolution maps (**a-e**) correspond to the different maps (8-12) depicted in **Supplementary Figure 5**.

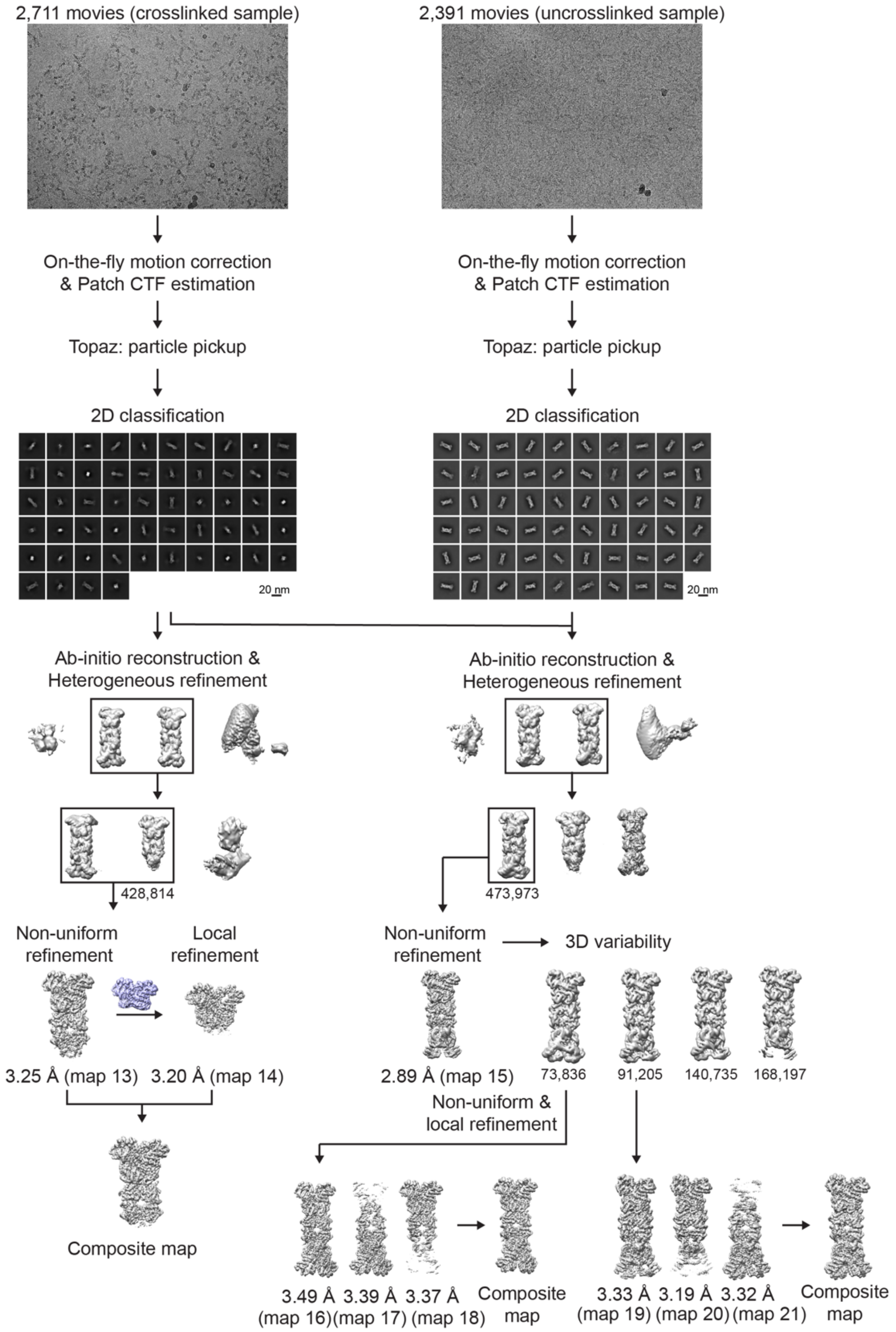

Supplementary Figure 7. Structure determination of DSR2-DSAD1 complex.

Workflow of the cryo-EM data processing to obtain the 3D reconstructions of DSR2-DSAD1 complex. Nine cryo-EM maps (labeled maps 13-21) were obtained for the whole structure.

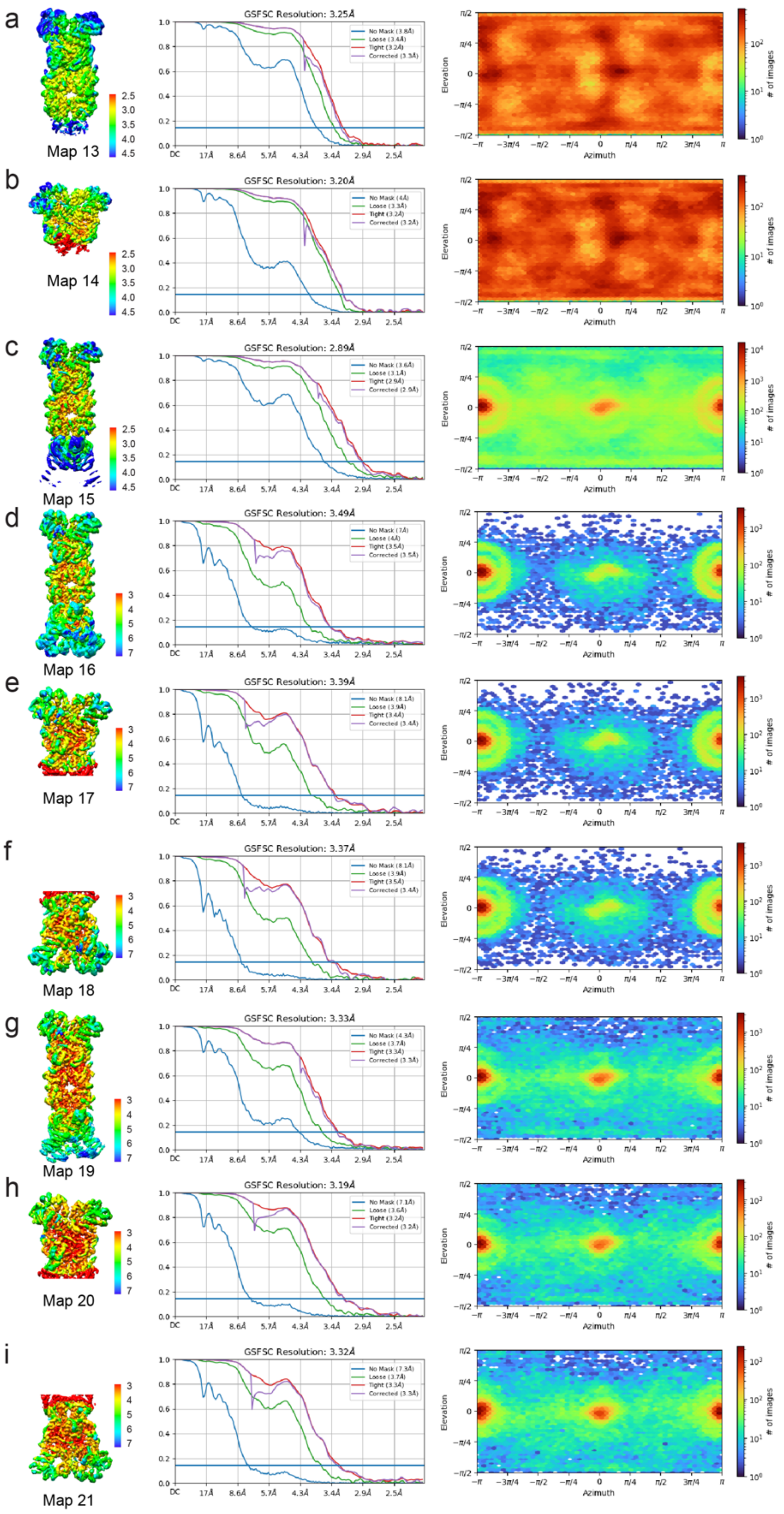

Supplementary Figure 8. The resolution analysis of DSR2-DSAD1 complex.

a-i Local resolution maps and Fourier shell correlation (FSC) curves of DSR2-DSAD1 complex. The local resolution maps (**a-i**) correspond to the different maps (13-21) depicted in **Supplementary Figure 7**.

Supplementary Table 1 | Cryo-EM data collection, refinement and validation statistics for apo DSR2 and DSR2-TTP-NAD$^+$ complex

	DSR2 (H171A) (crosslinked) (EMDB-38872) (PDB 8Y34)	(EMDB-38824) (PDB 8Y13)	DSR2 (H171A)-TTP-NAD$^+$ (EMDB-39925) (PDB 8ZC9)
**Data collection and processing**			
Magnification	130,000×		105,000×
Voltage (kV)	300		300
Electron exposure (e–/Å^2)	50		50
Defocus range (μm)	-1 – -1.5		-1.5 – -2.0
Pixel size (Å)	0.92		1.2
Symmetry imposed	C1		C1
Final particle images (no.)	86,488	46,211	
Map resolution (Å)	3.11	3.18	3.14
FSC threshold	0.143	0.143	0.143
**Refinement**			
Model resolution (Å)	3.3	3.4	3.4
FSC threshold	0.5	0.5	0.5
Map sharpening *B* factor (Å^2)	61.3	51.9	59.6
Model composition			
Non-hydrogen atoms	15869	31736	35350
Protein residues	1902	3804	4224
Ligands	0	0	4
B factors (Å^2)			
Protein	77.29	125.08	126.41
Ligand			106.55
R.m.s. deviations			
Bond lengths (Å)	0.004	0.004	0.003
Bond angles (°)	0.744	0.704	0.621
Validation			
MolProbity score	2.71	2.7	2.67
Clashscore	17.08	16.23	27.38
Poor rotamers (%)	5.49	4.55	2.98
Ramachandran plot			
Favored (%)	93.68	91.84	94.40
Allowed (%)	6.06	7.73	5.46
Disallowed (%)	0.27	0.43	0.14

Supplementary Table 2 | Cryo-EM data collection, refinement and validation statistics for DSR2-DSAD1 complex

	DSR2-DSAD1 (crosslinked) (EMDB-38889) (PDB 8Y3M)	DSR2-DSAD1 (merged) (EMDB-38902) (PDB 8Y3W)	(EMDB-38907) (PDB 8Y3Y)
**Data collection and processing**			
Magnification	81,000×	81,000×	
Voltage (kV)	300	300	
Electron exposure (e–/Å^2)	50	50	
Defocus range (μm)	-1.5 – -2	-1.5 – -2	
Pixel size (Å)	1.0773	1.0773	
Symmetry imposed	C1	C1	C1
Final particle images (no.)	428,814	73,836	91,205
Map resolution (Å)	3.25	3.49	3.33
FSC threshold	0.143	0.143	0.143
**Refinement**			
Model resolution (Å)	3.3	3.7	3.5
FSC threshold	0.5	0.5	0.5
Map sharpening *B* factor (Å^2)	140.9	89.6	84.2
Model composition			
Non-hydrogen atoms	16793	33586	33586
Protein residues	2017	4034	4034
Ligands	0	0	0
B factors (Å^2)			
Protein	96.72	119.19	108.10
Ligand			
R.m.s. deviations			
Bond lengths (Å)	0.003	0.005	0.004
Bond angles (°)	0.851	0.935	0.902
Validation			
MolProbity score	2.71	3.08	2.98
Clashscore	10.37	19.33	17.12
Poor rotamers (%)	7.39	7.28	7.63
Ramachandran plot			
Favored (%)	91.31	86.11	89.13
Allowed (%)	8.19	13.13	10.20
Disallowed (%)	0.5	0.75	0.68
