# Supplementary material for: The structural basis of the activation and inhibition of DSR2 NADase by phage proteins
Source: Nat Commun. 2024 Jul 23;15:6185. doi: 10.1038/s41467-024-50410-0 (PMC11263360; doi:10.1038/s41467-024-50410-0)
Supplement: Supplementary file 6 — Description of Additional Supplementary Files [file 41467_2024_50410_MOESM6_ESM.pdf]

**File name: Supplementary Movie 1**

3D variability analysis of DSR2 homodimer.

**File name: Supplementary Movie 2**

3D variability analysis of DSR2-TTP-$NAD^+$ complex.

**File name: Supplementary Movie 3**

3D variability analysis of DSR2-DSAD1 complex.

**File name: Supplementary Data 1**

The Sequences of oligonucleotides (primers) used in this study are listed as Supplementary Data.
